# Focal Adhesion Kinase (FAK) tyrosine 397E mutation restores the vascular leakage defect in endothelium‐specific FAK‐kinase dead mice

**DOI:** 10.1002/path.4911

**Published:** 2017-06-01

**Authors:** Annika N Alexopoulou, Delphine M Lees, Natalia Bodrug, Tanguy Lechertier, Isabelle Fernandez, Gabriela D'Amico, Matthew Dukinfield, Silvia Batista, Bernardo Tavora, Bryan Serrels, Kairbaan Hodivala‐Dilke

**Affiliations:** ^1^ Department of Molecular Oncology, BSRC Alexander Fleming Athens Greece; ^2^ Centre for Tumour Biology, Barts Cancer Institute Queen Mary University of London London UK; ^3^ Platform of Expertise for Rare Diseases Paris‐Sud Le Kremlin‐Bicêtre France; ^4^ Division of Cancer Therapeutics, Institute of Cancer Research Sutton UK; ^5^ Laboratory of Systems Cancer Biology Rockefeller University New York USA; ^6^ Cancer Research UK Edinburgh Centre University of Edinburgh Edinburgh UK

**Keywords:** tumour angiogenesis, focal adhesion kinase

## Abstract

Focal adhesion kinase (FAK) inhibitors have been developed as potential anticancer agents and are undergoing clinical trials. In vitro activation of the FAK kinase domain triggers autophosphorylation of Y397, Src activation, and subsequent phosphorylation of other FAK tyrosine residues. However, how FAK Y397 mutations affect FAK kinase‐dead (KD) phenotypes in tumour angiogenesis in vivo is unknown. We developed three Pdgfb‐iCre^ert^‐driven endothelial cell (EC)‐specific, tamoxifen‐inducible homozygous mutant mouse lines: FAK wild‐type (WT), FAK KD, and FAK double mutant (DM), i.e. KD with a putatively phosphomimetic Y397E mutation. These ECCre+;FAK^WT^ ^/^ ^WT^, ECCre+;FAK^KD^ ^/^ ^KD^ and ECCre+;FAK^DM^ ^/^ ^DM^ mice were injected subcutaneously with syngeneic B16F0 melanoma cells. Tumour growth and tumour blood vessel functions were unchanged between ECCre+;FAK^WT^ ^/^ ^WT^ and ECCre−;FAK^WT^ ^/^ ^WT^ control mice. In contrast, tumour growth and vessel density were decreased in ECCre+;FAK^KD^ ^/^ ^KD^ and ECCre+;FAK^DM^ ^/^ ^DM^ mice, as compared with Cre − littermates. Despite no change in the percentage of perfused vessels or pericyte coverage in either genotype, tumour hypoxia was elevated in ECCre+;FAK^KD^ ^/^ ^KD^ and ECCre+;FAK^DM^ ^/^ ^DM^ mice. Furthermore, although ECCre+;FAK^KD^ ^/^ ^KD^ mice showed reduced blood vessel leakage, ECCre+;FAK^DM^ ^/^ ^DM^ and ECCre−;FAK^DM^ ^/^ ^DM^ mice showed no difference in leakage. Mechanistically, fibronectin‐stimulated Y397 autophosphorylation was reduced in Cre+;FAK^KD^ ^/^ ^KD^ ECs as compared with Cre+;FAK^WT^ ^/^ ^WT^ cells, with no change in phosphorylation of the known Src targets FAK‐Y577, FAK‐Y861, FAK‐Y925, paxillin‐Y118, p130Cas‐Y410. Cre+;FAK^DM^ ^/^ ^DM^ ECs showed decreased Src target phosphorylation levels, suggesting that the Y397E substitution actually disrupted Src activation. Reduced VE‐cadherin‐pY658 levels in Cre+;FAK^KD^ ^/^ ^KD^ ECs were rescued in Cre+FAK^DM^ ^/^ ^DM^ ECs, corresponding with the rescue in vessel leakage in the ECCre+;FAK^DM^ ^/^ ^DM^ mice. We show that EC‐specific FAK kinase activity is required for tumour growth, angiogenesis, and vascular permeability. The ECCre+;FAK^DM/DM^ mice restored the KD‐dependent tumour vascular leakage observed in ECCre+;FAK^KD/KD^ mice in vivo. This study opens new fields in in vivo FAK signalling. © 2017 The Authors. *The Journal of Pathology* published by John Wiley & Sons Ltd on behalf of Pathological Society of Great Britain and Ireland.

## Introduction

Tumour growth and spread require angiogenesis. Understanding the molecular mechanisms by which tumour blood vessels develop *in vivo* is necessary for a better understanding of the drivers of tumour angiogenesis. Focal adhesion kinase (FAK) is a 125‐kDa non‐receptor tyrosine kinase involved in tumour angiogenesis 1, 2. Upon activation by various stimuli, including integrins and growth factors, FAK autophosphorylation at Y397 leads to binding and activation of Src, which in turn phosphorylates other FAK residues, including Y576, Y577, Y861, and Y925 3, 4. Apart from its role as a kinase, FAK also plays a role as a scaffolding protein, with binding sites for several proteins, making FAK a central player in cellular processes that include adhesion, migration, invasion, cell proliferation, and apoptosis 4, 5.

We have previously shown that Pdgfb‐iCre^ert^‐inducible endothelial cell (EC)‐specific homozygous deletion of FAK leads to reduced primary tumour growth and angiogenesis 2. Other reports have indicated that hemizygous EC‐specific FAK kinase‐dead (KD) mice show no effect on primary tumour growth, but show reduced vascular endothelial growth factor (VEGF)‐stimulated vascular permeability 6 and metastasis 7. These data have supported the development of pharmacological FAK kinase inhibitors. Indeed, inhibition of FAK kinase activity reduced vascular permeability and metastasis 6, 7. Similar results were obtained with Src inhibitors and in Src‐null mice 8. FAK and Src may act in concert to regulate vascular permeability and metastasis 7. Mice that are haploinsufficient for FAK expression show increased tumour growth and angiogenesis, indicating a dose‐dependent role for FAK 9, and this has raised the notion that homozygous FAK KD animals may show a different tumour angiogenic phenotype *in vivo*.

To further delineate which domains of FAK regulate these phenotypes *in vivo*, we have recently generated a series of endothelial‐specific inducible mutant FAK knockin mice 10. In this study, we used the FAK‐K454R mutant, which is KD 11, and a double mutant (DM) that carries both the KD mutation and a glutamate substitution of Y397 (Y397E), in order to test the requirement for FAK autophosphorylation and Src recruitment. Unlike previously published FAK‐mutant mice, in our system, upon tamoxifen induction, Cre recombinase is activated in ECs, leading to deletion of endogenous FAK and expression of a myc‐tagged knockin FAK under control of the Rosa26 (R26) promoter, in both alleles. Thus, after recombination in ECs, the endogenous FAK is replaced by the myc‐tagged FAK, expressed under control of the R26 promoter, at a dose similar to that of endogenous FAK. As the knockin FAK is expressed under control of the R26 promoter, our system does not reflect any changes due to FAK promoter regulation 10, as other models have 6, 7, 12.

## Materials and methods

Full information on the methods and reagents used in this study are available in supplementary materials and methods.

### Mice

The work described was approved by the QMUL Ethics Committee. To study the effect of inducible EC‐specific FAK mutations *in vivo*, we used a knockout/knockin system whereby endogenous mouse FAK was deleted and myc‐tagged chicken FAK [wild‐type (WT) or mutant] was expressed under control of the R26 promoter upon tamoxifen induction. We developed Pdgfb‐iCre^ert^;FAK^fl/fl^;R26FAK^KD/KD^ mice (FAK KD) and Pdgfb‐iCre^ert^;FAK^fl/fl^;R26FAK^DM/DM^ DM mice, in which Y397 has been additionally mutated to a glutamate. Pdgfb‐iCre^ert^;FAK^fl/fl^;R26FAK^WT/WT^ mice expressed WT FAK, and were used to validate the system. The generation and characterization of these mice, together with proof of chicken FAK knockin, have been described previously 10.

### Tumour growth assays

Mice aged 12–16 weeks were given tamoxifen (Sigma, Irvine, UK) via intraperitoneal injection on two consecutive days; 3 days later, 10^6^ B16F0 mouse melanoma cells (ATCC) were injected subcutaneously into the flanks of these mice. Mice were given a tamoxifen‐containing diet (Tamoxifen400; Harlan Laboratories, Blackthorn, Bicester, UK), from the day of the second tamoxifen injection until the end of the experiment. Tumour sizes were measured with digital calipers on alternate days. Mice were killed by day 18 after tumour cell injection. Tumour volumes were calculated with the formula volume = length × width^2^ × 0.52. Tumours were snap‐frozen in isopentane chilled in liquid nitrogen. Four tumour growth experiments per genotype were performed.

### Tumour immunohistochemistry

Counting and image analysis were performed with an epifluorescence Zeiss Axioplan Microscope (Zeiss, Munich, Germany). High‐quality images were captured with a Carl Zeiss LSM 710 confocal microscope.

#### Blood vessel density

Frozen sections were fixed with cold acetone for 10 min, blocked with 1% bovine serum albumin (BSA) in phosphate‐buffered saline (PBS), incubated with a rat anti‐endomucin antibody (clone V.7c7, 1:200; Santa Cruz Biotechnology, Dallas, TX, USA) overnight at 4 °C, washed in PBS, and then incubated for 60 min at room temperature with an Alexa Fluor‐labelled anti‐rat antibody (A‐21208, 1:200; Invitrogen, Loughborough, UK). Tumour blood vessels were counted across entire midline sections, and the numbers were expressed as vessels per mm^2^ of tumour section.

#### Functional vessel analysis

Mice with size‐matched tumours were tail vein‐injected with 100 µl of 200 µg/ml phycoerythrin (PE)‐conjugated anti‐PECAM antibody (clone 390, neat; Biolegend, San Diego, CA, USA) 10 min before they were killed. To measure the percentage of functional vessels, tumour sections were stained for endomucin to identify all tumour blood vessels, and the percentage of endomucin‐positive vessels that were PE‐PECAM‐perfused was calculated.

#### Pericyte coverage

Frozen tumour sections were fixed with cold acetone, blocked in 1% BSA in PBS, washed, incubated overnight at 4 °C with a rabbit anti‐NG2 antibody (AB5320, 1:300; Millipore, Watford, Hertfordshire, UK) and a rat anti‐endomucin antibody (V.7C7, 1:200; Santa Cruz Biotechnology), washed, incubated with an Alexa Fluor‐488 anti‐rabbit antibody and an Alexa Fluor‐546 anti‐rat antibody (A21206 and A11081, both 1:200; Invitrogen) for 1 h at room temperature, and mounted. The percentage of endomucin‐positive vessels with NG2‐positive cells associated was calculated.

#### Vascular permeability

Mice with size‐matched tumours were tail vein‐injected with 100 µl of 4 mg/ml Hoechst 33342 1 min before being killed, and with PE‐conjugated anti‐PECAM antibody as described above. Frozen tumour sections were fixed in cold acetone and mounted. Pictures of 5–10 fields were taken with a × 20 objective lens and analysed with Image J. Hoechst‐positive areas divided by PE‐PECAM‐positive areas were calculated to give permeability ratios.

#### Hypoxia analysis

Pimonidazole‐HCl (Hypoxyprobe, Burlington, MA, USA) at 60 mg/kg body weight was intraperitoneally injected 1 h before mice were killed. Frozen tumour sections were acetone‐fixed, incubated overnight at 4 °C with a fluorescein isothiocyanate‐conjugated mouse anti‐pimonidazole antibody (4.3.11.3, 1:10; Hypoxyprobe), and then washed and mounted. Sections were analysed at × 10 objective magnification, with 8–10 pictures per case. The percentage of hypoxic area over the total section area was calculated.

### EC preparation

Mouse lung ECs were isolated from three adult mice per genotype, as previously described 13. The purity of ECs prepared with our method has been established 2. Anti‐ICAM2 clone IC2/4 (3C4), (Abd Serotec, Martinsried, Germany); anti‐VECAD clone 11D4.1; (BD Biosciences San Joe, CA, USA) were used to assess EC purity by flow cytometry on a Becton Dickinson, Oxford, UK, FACSCalibur instrument. As a negative control, IgG‐matched isotypes were used (data not shown). Cells were immortalized by two rounds of infection with polyoma virus middle T 14. To ensure maximal Cre‐induced recombination, lung ECs isolated from Cre‐positive mice were transfected with a Cre‐encoding plasmid (pCAG‐Cre‐IRES2‐GFP; Addgene, Cambridge, MA, USA 26646), and cells isolated from Cre‐negative mice were transfected with the corresponding plasmid backbone (pCAG‐GFP; Addgene 11150). Alternatively, immortalized cells were maintained in medium containing 500 nm 4‐hydroxytamoxifen from day 4 after isolation.

### FAK immunoprecipitation and myc‐tag western blotting

RIPA lysates from immortalized lung ECs or mouse hearts were used. A Dynabeads protein G immunoprecipitation kit (Invitrogen) was used according to the manufacturer's instructions. FAK was immunoprecipitated with anti‐FAK antibody (clone C20, 1:20; Santa Cruz Biotechnology) that recognizes all FAK in the cell (i.e. both mouse and chicken FAK). The bound protein was eluted and loaded onto Tris‐acetate polyacrylamide gels. Separated proteins were transferred to nitrocellulose, blocked, and probed with anti‐myc tag antibody (clone 9E10, 1:1000; Abcam, Cambridge, UK) or anti‐FAK antibody (mouse anti‐FAK, 1:1000; 610088; BD Transduction Laboratories, Oxford, UK) as a control.

## Results

**Figure 1:**
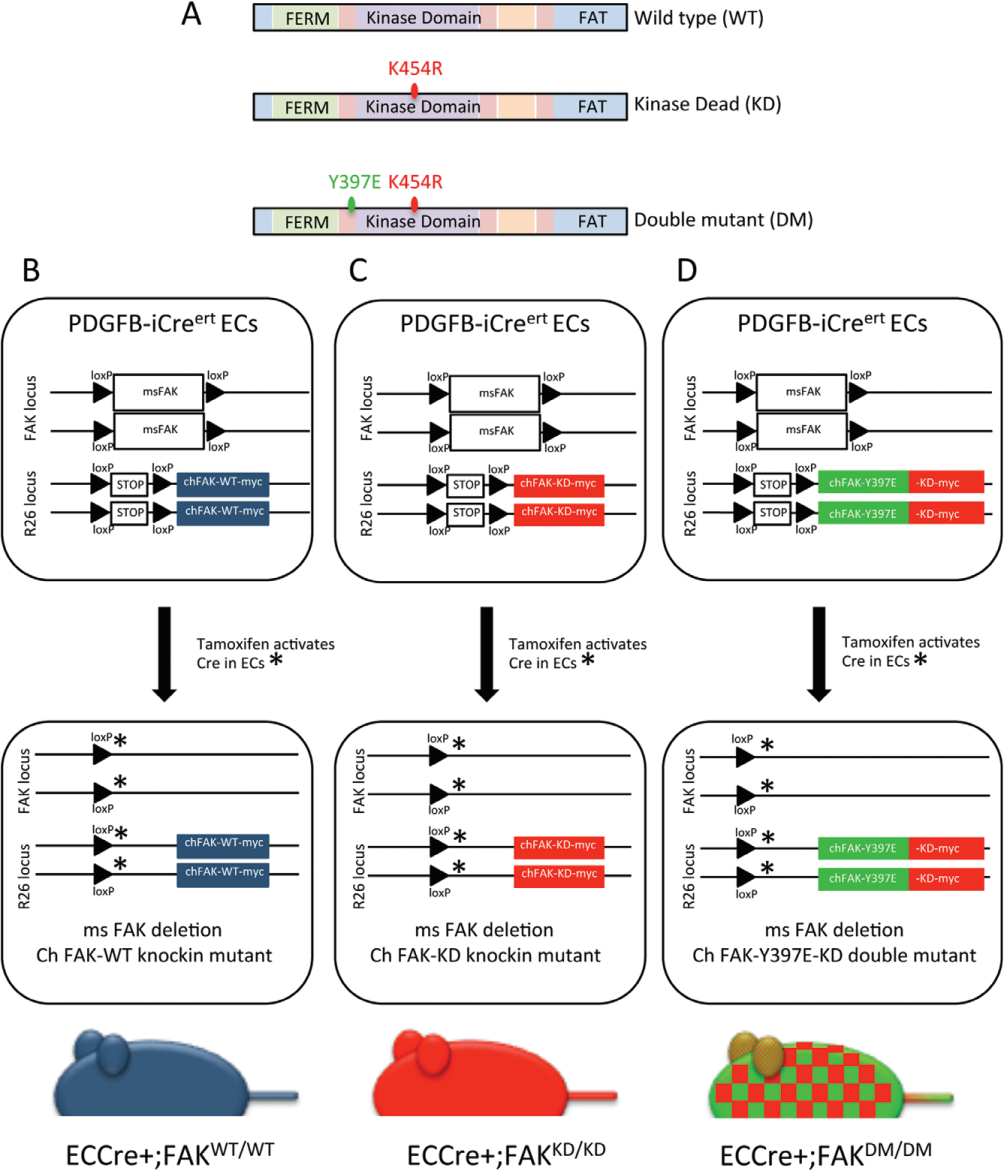
Generation of Pdgfb‐iCre^ert^;FAK^fl/fl^;R26FAK^WT/WT^, Pdgfb‐iCre^ert^;FAK^fl/fl^;R26FAK^KD/KD^ and Pdgfb‐iCre^ert^;FAK^fl/fl^;R26FAK^DM/DM^ mice. (A) Schematic representation of WT FAK, the K454R KD mutant FAK and the DM harbouring both the K454R and Y397E mutations. (B–D) Schematic representations of Pdgfb‐iCre^ert^;FAK^fl/fl^;R26FAK^WT/WT^, Pdgfb‐iCre^ert^;FAK^fl/fl^;R26FAK^KD/KD^ and Pdgfb‐iCre^ert^;FAK^fl/fl^;R26FAK^DM/DM^ mouse genotypes. Endogenous FAK is floxed, and knockin chicken FAK mutants were produced as homozygotes. Administration of tamoxifen induces endogenous mouse FAK deletion and WT, KD or DM knockin chicken FAK expression, to generate (B) ECCre + FAK^WT/WT^, (C) ECCre + FAK^KD/KD^, and (D) ECCre + FAK^DM/DM^ mice, respectively.

We generated mouse lines to express a knockin WT chicken FAK (Pdgfb‐iCre^ert^;FAK^fl/fl^;R26FAK^WT/WT^), a KD K454R mutant chicken FAK (Pdgfb‐iCre^ert^;FAK^fl/fl^;R26FAK^KD/KD^), and a DM Y397E/K454R chicken FAK (Pdgfb‐iCre^ert^;FAK^fl/fl^;R26FAK^DM/DM^) (Figure 1A). Tamoxifen treatment induced deletion in ECs of the endogenous mouse FAK, with simultaneous expression of myc‐tagged knockin chicken FAK. These mice are referred to as ECCre+;FAK^WT/WT^, ECCre+;FAK^KD/KD^, and ECCre+;FAK^DM/DM^, respectively (Figure 1B–D). Tamoxifen‐treated Pdgfb‐iCre^ert^‐negative littermates were used as negative controls. WT chicken FAK has been shown to fully substitute for mouse FAK in other systems 15, 16, and sequence homology within the nuclear localization motif and nuclear export signal sequences of mouse and chicken FAK suggests that the nuclear localizations of mouse and chicken FAK do not differ 17, 18.

**Figure 2:**
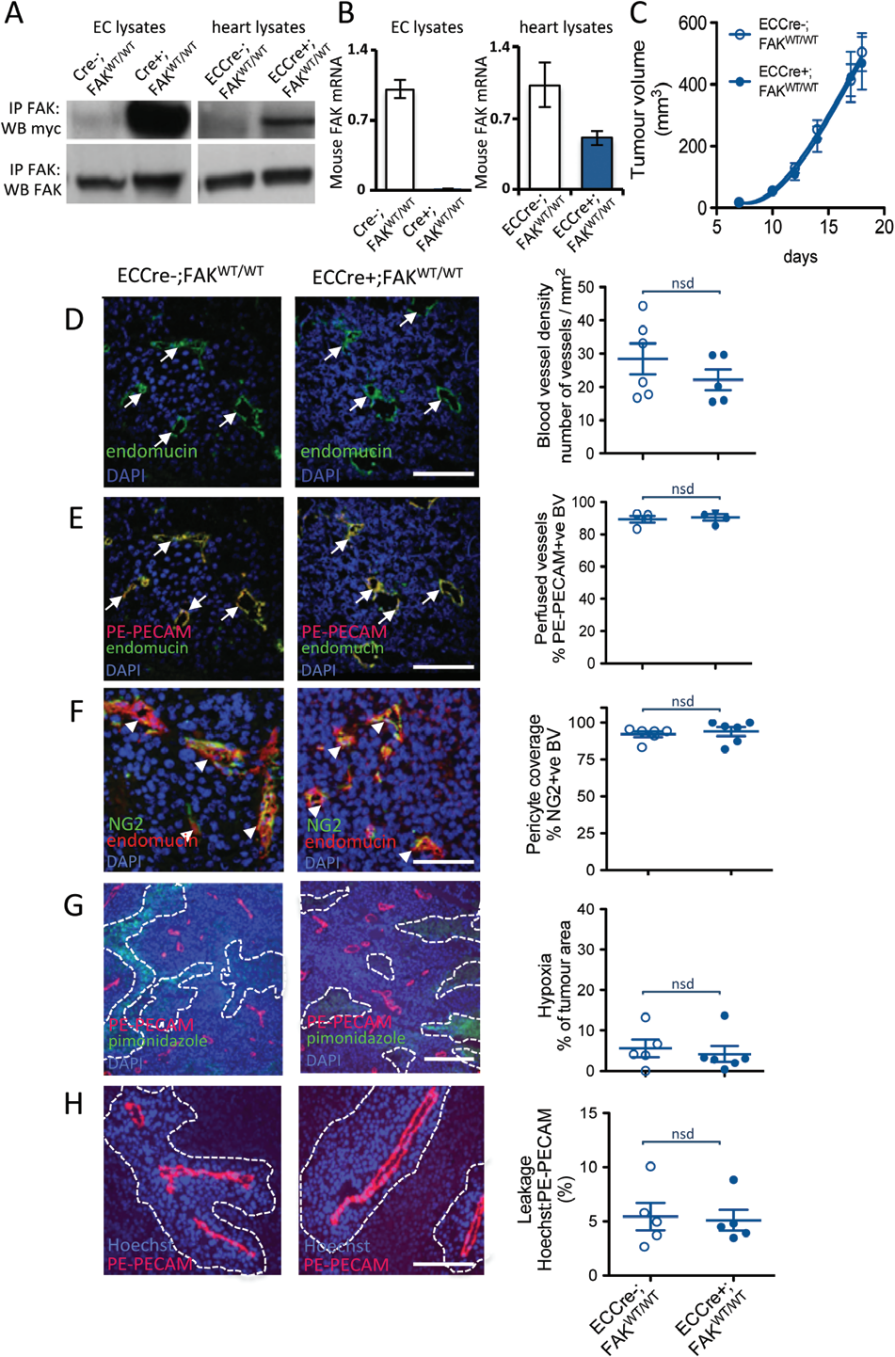
ECCre+;FAK^WT/WT^ mice and ECCre−;FAK^WT/WT^ mice show similar tumour growth and angiogenesis. (A) Immunoprecipitation (IP) for FAK and Western blotting (WB) for the myc‐tag demonstrate increased myc expression in Cre+;FAK^WT/WT^ EC lysates as compared with Cre−;FAK^WT/WT^ control lysates, indicating WT chicken FAK knockin expression in the Cre + ECs. Knockin was also verified in vivo, by similar analysis of fresh heart lysates made from tamoxifen‐treated PdgfbCre+;FAK^fl/fl^;R26FAK^WT/WT^ (ECCre + FAK^WT/WT^) mice, but not PgdfbCre−;FAK^fl/fl^;R26FAK^WT/WT^ (ECCre−;FAK^WT/WT^) mice. WB for FAK indicated that the knockin was expressed at a similar level to endogenous FAK. The images shown are representative of five experiments. (B) qPCR analysis of mouse FAK shows reduced endogenous mouse FAK mRNA in Cre+;FAK^WT/WT^ EC lysates and ECCre+;FAK^WT/WT^ fresh heart lysates, as compared with Cre − controls. The graphs represent mean values ± standard errors of the mean (SEMs) relative to the glyceraldehyde‐3‐phosphate dehydrogenase gene; n = 5 replicates. (C) Tumour growth was not statistically different between ECCre−;FAK^WT/WT^ and ECCre+;FAK^WT/WT^ mice injected subcutaneously with 1 million B16F0 cells. The graph represents means ± SEMs; n = 13 (ECCre−;FAK^WT/WT^) and n = 15 (ECCre+;FAK^WT/WT^). (D–H) Sections of end‐stage tumours grown in ECCre−;FAK^WT/WT^ and ECCre+;FAK^WT/WT^ mice were analysed by immunohistochemistry for: (D) blood vessel density (arrows, endomucin‐positive blood vessels); (E) blood vessel perfusion (arrows, PE‐PECAM/endomucin‐positive blood vessels); (F) pericyte coverage of blood vessels (arrowheads, NG2/endomucin‐positive blood vessels); (G) tumour hypoxia (dotted lines delineate pimonidazole‐positive tumour areas); and (H) blood vessel leakage (dotted lines delineate Hoechst‐positive areas around PE‐PECAM‐positive blood vessels). No significant differences in any of these features was observed between tumours grown in ECCre−;FAK^WT/WT^ and ECCre+;FAK^WT/WT^ mice. Representative immunofluorescence images are shown. Scale bars: 100 µm in (D), (E), (F) and (H); 250 µm in (G). Quantification is given as scatter plots: points represent mean values for each mouse, with means ± SEMs for each genotype. n = 4–6 mice per genotype. BV, blood vessels; DAPI, 4′,6‐diamidino‐2‐phenylindole; nsd, no statistically significant difference.

ECs were isolated from Cre‐negative FAK^fl/fl^;R26FAK^WT/WT^ and Pdgfb‐iCre^ert^;FAK^fl/fl^;R26FAK^WT/WT^ mice, and treated with tamoxifen *in vitro* to generate Cre−;FAK^WT/WT^ and Cre+;FAK^WT/WT^ cells. As antibodies that distinguish between mouse and chicken FAK do not currently exist, we used the myc‐tag to identify knockin FAK expression. Immunoprecipitation of EC lysates with an antibody that recognizes both mouse and chicken FAK, followed by western blotting for myc‐tag, showed that the myc‐tagged knockin FAK was expressed in Cre+;FAK^WT/WT^ but not in Cre−;FAK^WT/WT^ ECs. To assess the expression of knockin FAK *in vivo*, fresh hearts were isolated from tamoxifen‐treated ECCre−;FAK^WT/WT^ and ECCre+;FAK^WT/WT^ mice and lysed. Immunoprecipitation of FAK followed by western blotting for myc‐tag showed that the myc‐tagged chicken FAK protein was expressed in whole heart tissue from ECCre+;FAK^WT/WT^ but not from Cre−;FAK^WT/WT^ mice (Figure 2A). Quantitative polymerase chain reaction (qPCR) analysis demonstrated loss of endogenous mouse FAK mRNA in Cre+;FAK^WT/WT^ but not Cre−;FAK^WT/WT^ ECs and hearts (Figure 2B). Together, these data indicate that the knockin WT chicken FAK was expressed and that the endogenous mouse FAK was deleted in ECs from ECCre+;FAK^WT/WT^ mice and *in vivo*.

Loss of EC FAK in adult mice reduces tumour growth and tumour angiogenesis 2. To show that WT chicken FAK could reproduce the effects of endogenous mouse FAK *in vivo*, we first examined tumour growth and angiogenesis in Pdgfb‐iCre^ert^;FAK^fl/fl^;R26FAK^WT/WT^ mice. ECCre−;FAK^WT/WT^ and ECCre+;FAK^WT/WT^ mice were injected subcutaneously with 1 million syngeneic mouse B16F0 melanoma cells, and tumour growth was measured over time. There was no difference in tumour growth between ECCre−;FAK^WT/WT^ and ECCre+;FAK^WT/WT^ mice (Figure 2C). The salient features of tumour blood vessels were then analysed histologically, including blood vessel density, blood vessel perfusion, pericyte coverage, tumour hypoxia, and blood vessel leakage. Blood vessel density (number of endomucin‐positive vessels per mm^2^ of age‐matched, size‐matched tumour sections) was unchanged between B16F0 tumours grown in ECCre−;FAK^WT/WT^ and ECCre+;FAK^WT/WT^ mice (Figure 2D). To measure blood vessel perfusion, mice were injected *ante mortem* via the tail vein with a PE‐labelled anti‐PECAM antibody (PE‐PECAM) to label perfused vessels. Tumour sections were then immunostained for endomucin to identify all vessels. The percentage of endomucin‐positive vessels also positive for PE‐PECAM was not significantly different between ECCre−;FAK^WT/WT^ and ECCre+;FAK^WT/WT^ mice (Figure 2E). To examine pericyte coverage of tumour blood vessels, which is indicative of their maturation, sections were double‐immunostained for the pericyte marker NG2 and the endothelial marker endomucin. The percentage of NG2‐positive blood vessels was similar between tumours grown in ECCre−;FAK^WT/WT^ and ECCre+;FAK^WT/WT^ mice (Figure 2F). Changes in tumour hypoxia constitute an indicator of aberrant tumour blood vessel development. Tumour hypoxia, assessed *in vivo* by *ante mortem* injection of pimonidazole and measurement of areas of pimonidazole‐positive tumour sections, did not differ between ECCre−;FAK^WT/WT^ and ECCre+;FAK^WT/WT^ mice (Figure 2G). To examine blood vessel leakage, mice were injected *ante mortem* with Hoechst dye and PE‐PECAM. Leakage was analysed by measuring the Hoechst area relative to the endothelial PE‐PECAM‐positive area, and showed no difference between ECCre−;FAK^WT/WT^ and ECCre+;FAK^WT/WT^ mice (Figure 2H). Together, these data indicate that EC‐specific WT myc‐tagged chicken FAK knockin has no significant effect on tumour growth or tumour blood vessels, and confirms that the chicken FAK knockin can rescue the deleted mouse FAK in this system.

**Figure 3:**
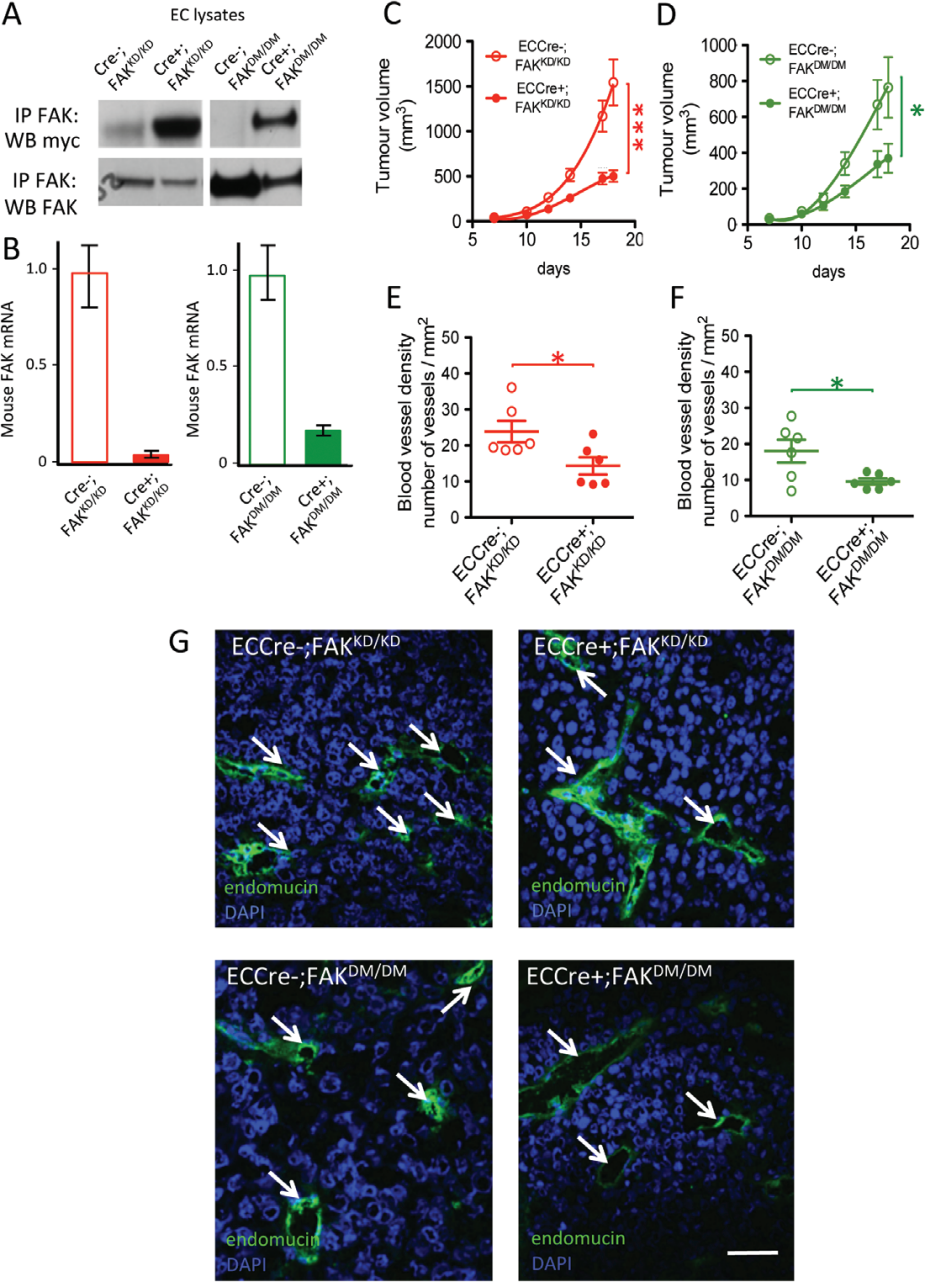
ECCre+;FAK^KD/KD^ mice show reduced tumour growth and associated angiogenesis, and this is not rescued in ECCre+;FAK^DM/DM^ mice. (A) Immunoprecipitation (IP) for FAK and Western blotting (WB) for the myc‐tag demonstrates increased myc‐tag expression in Cre+;FAK^KD/KD^ EC lysates as compared with Cre−;FAK^KD/KD^ control lysates, and in Cre+;FAK^DM/DM^ EC lysates as compared with Cre−;FAK^DM/DM^ control lysates, indicating mutant FAK knockin expression in Cre + ECs. FAK WB shows similar expression levels of endogenous and knockin FAK. Blots are representative of three experimental repeats. (B) qPCR analysis of mouse FAK shows reduced endogenous mouse FAK mRNA levels in Cre+;FAK^KD/KD^ EC lysates as compared with Cre−;FAK^KD/KD^ control lysates, and in Cre+;FAK^DM/DM^ EC lysates as compared with Cre−;FAK^DM/DM^ control lysates. Graphs represent mean values ± standard errors of the mean, relative to the glyceraldehyde‐3‐phosphate dehydrogenase gene; n = 2 experimental repeats. (C, D) B16F0 tumour cells were injected subcutaneously into (C) ECCre−;FAK^KD/KD^ (n = 14) and ECCre+;FAK^KD/KD^ (n = 15) mice, or (D) ECCre−;FAK^DM/DM^ (n = 13) and ECCre+;FAK^DM/DM^ (n = 16), mice and tumour growth was monitored over time. (E, F) Endomucin‐positive blood vessel density was quantified in midline tumour sections from tumours grown in (E) ECCre−;FAK^KD/KD^ and ECCre+;FAK^KD/KD^ mice, or (F) ECCre−;FAK^DM/DM^ and ECCre+;FAK^DM/DM^ mice. n = 6 tumours per genotype. (G) Immunofluorescence detection of endomucin‐positive blood vessels in tumours grown in ECCre−;FAK^KD/KD^ and ECCre+;FAK^KD/KD^ mice, or ECCre−;FAK^DM/DM^ and ECCre+;FAK^DM/DM^ mice. Arrows: endomucin‐positive blood vessels. Scale bar: 50 µm. Student's t‐test: *p < 0.05, ***p < 0.001. DAPI, 4′,6‐diamidino‐2‐phenylindole.

We next sought to compare the *in vivo* effect of EC‐specific FAK KD mutation, alone or in combination with the phosphomimetic Y397E mutation (in DM mice). To confirm the endogenous FAK deletion and the knockin (KD or DM) FAK expression, Cre−;FAK^KD/KD^, Cre+;FAK^KD/KD^, Cre−;FAK^DM/DM^ and Cre+;FAK^DM/DM^ mouse lung ECs were isolated and lysed. Immunoprecipitation of FAK followed by western blotting for myc‐tag showed that the FAK KD or the FAK DM myc‐tagged proteins were expressed in Cre+;FAK^KD/KD^ and Cre+;FAK^DM/DM^ ECs but not in Cre‐negative controls (Figure 3A). qPCR analysis confirmed loss of endogenous mouse FAK mRNA in Cre+;FAK^KD/KD^ and Cre+;FAK^DM/DM^ ECs, but not in Cre‐negative controls (Figure 3B). Immunofluorescence analysis demonstrated that FAK and myc‐tag colocalized in cultured ECs in Cre+;FAK^WT/WT^, Cre+;FAK^KD/KD^ and Cre+;FAK^DM/DM^ cells (supplementary material, Figure S1).

ECCre−;FAK^KD/KD^, ECCre+;FAK^KD/KD^, ECCre−;FAK^DM/DM^ and ECCre+;FAK^DM/DM^ mice were injected subcutaneously with B16F0 melanoma cells. Tumour growth was reduced in ECCre+;FAK^KD/KD^ mice as compared with ECCre−;FAK^KD/KD^ controls (Figure 3C). Tumour growth also was reduced in ECCre+;FAK^DM/DM^ mice as compared with ECCre−;FAK^DM/DM^ mice (Figure 3D). Immunohistochemical analysis of end‐stage midline tumour sections indicated lower endomucin‐positive blood vessel densities in ECCre+;FAK^KD/KD^ and ECCre+;FAK^DM/DM^ mice than in ECCre−;FAK^KD/KD^ and ECCre−;FAK^DM/DM^ mice, respectively (Figure 3E–G).

**Figure 4:**
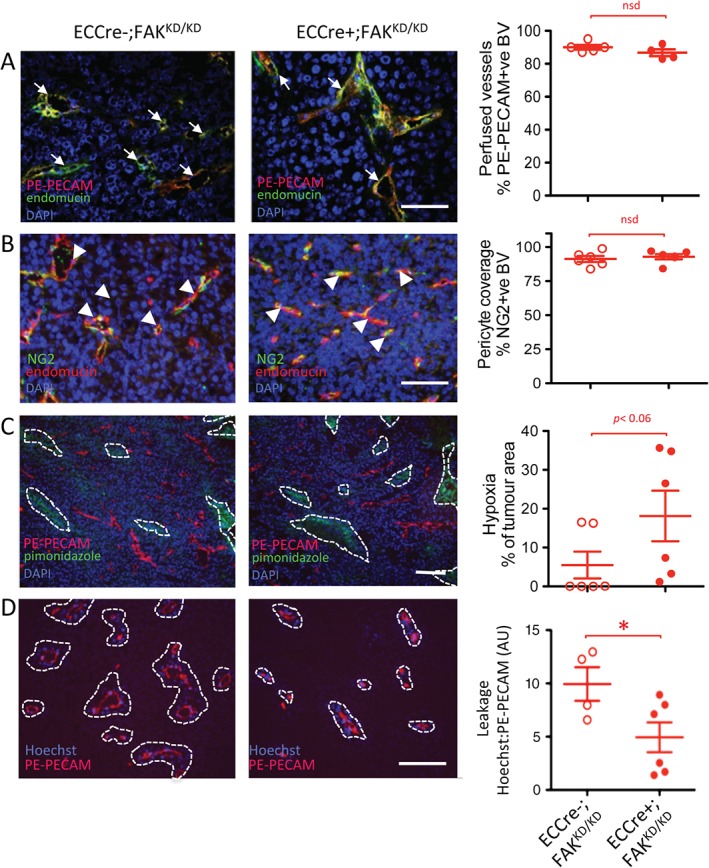
Reduced tumour vasculature leakage and enhanced tumour hypoxia in ECCre+;FAK^KD/KD^ mice. Sections of end‐stage tumours grown in ECCre−;FAK^KD/KD^ and ECCre+;FAK^KD/KD^ mice were analysed by immunohistochemistry for: (A) blood vessel perfusion (arrows, PE‐PECAM/endomucin‐positive blood vessels); (B) pericyte coverage of blood vessels (arrowheads, NG2/endomucin‐positive blood vessels); (C) tumour hypoxia (dotted lines delineate pimonidazole‐positive tumour areas); and (D) blood vessel leakage (dotted lines delineate Hoechst‐positive areas around PE‐PECAM‐positive blood vessels). Representative immunofluorescence images are shown. Quantification is given in scatter plots: points represent mean values for individual mice, with means ± standard errors of the mean for each genotype. Scale bars: 100 µm in (A), (B), and (D); 250 µm in (C). Student's t‐test: *p < 0.05. n = 4–6 mice per genotype. BV, blood vessels; DAPI, 4′,6‐diamidino‐2‐phenylindole; nsd, no statistically significant difference.

As in the ECCre−;FAK^WT/WT^ and ECCre+;FAK^WT/WT^ mice described above, we next analysed blood vessels in sections of tumours grown in ECCre−;FAK^KD/KD^ and ECCre+;FAK^KD/KD^ mice. No differences in blood vessel perfusion (Figure 4A) or NG2 pericyte association (Figure 4B) were observed between blood vessels of tumours grown in ECCre−;FAK^KD/KD^ and ECCre+;FAK^KD/KD^ mice. Tumour hypoxia, measured by relative pimonidazole levels, showed a non‐significant (*p* < 0.06) increase in tumours grown in ECCre+;FAK^KD/KD^ mice (Figure 4C), correlating with the reduced blood vessel density in these mice. Hoechst blood vessel leakage was decreased in tumours grown in ECCre+;FAK^KD/KD^ mice as compared with ECCre−;FAK^KD/KD^ controls (Figure 4D), suggesting that the KD mutation was sufficient to affect leakage.

**Figure 5:**
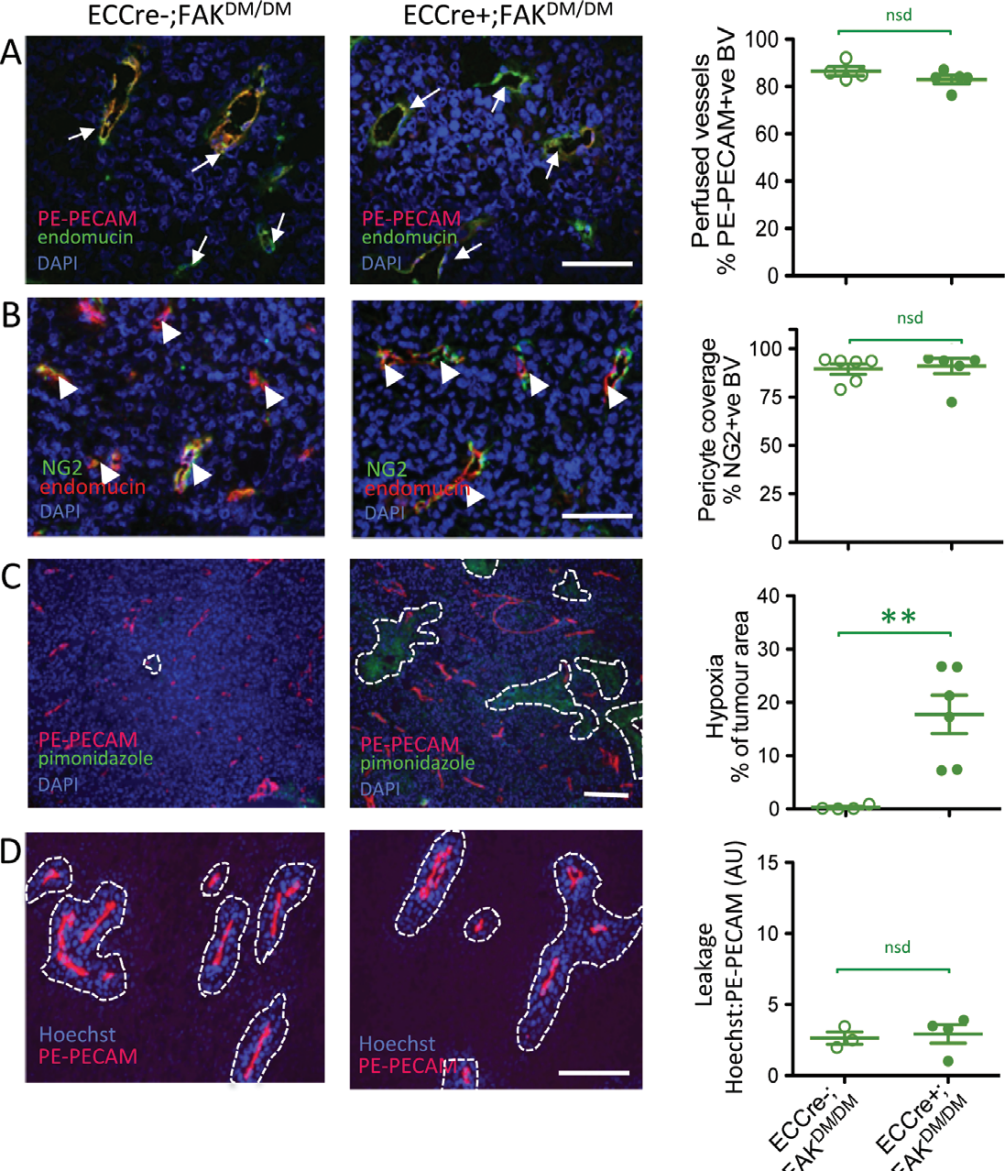
Tumour vascular leakage is not affected in ECCre+;FAK^DM/DM^ mice. Sections of end‐stage tumours grown in ECCre−;FAK^DM/DM^ and ECCre+;FAK^DM/DM^ mice were analysed by immunohistochemistry for: (A) blood vessel perfusion (arrows, PE‐PECAM/endomucin‐positive blood vessels); (B) pericyte coverage of blood vessels (arrowheads, NG2/endomucin‐positive blood vessels); (C) tumour hypoxia (dotted lines delineate pimonidazole‐positive tumour areas); and (D) blood vessel leakage (dotted lines delineate Hoechst‐positive areas around PE‐PECAM‐positive blood vessels). Representative immunofluorescence images are shown. Quantification is given in scatter plots: points represent mean values for individual mice, with means ± standard errors of the mean for each genotype. Scale bars: 100 µm in (A), (B), and (D); 250 µm in (C). Student's t‐test: **p < 0.01. n = 3–6 mice per genotype. BV, blood vessels; DAPI, 4′,6‐diamidino‐2‐phenylindole; nsd, no statistically significant difference.

The effect of a FAK Y397E mutation in combination with the KD mutation was then studied, in ECCre+;FAK^DM/DM^ mice. Immunohistochemical analysis of tumour sections from ECCre−;FAK^DM/DM^ and ECCre+;FAK^DM/DM^ mice again showed no significant differences in blood vessel perfusion or NG2‐positive pericyte association (Figure 5A, B), similarly to the results shown in Figure 4A, B. Analysis of tumour hypoxia showed that pimonidazole levels were higher in tumours grown in ECCre+;FAK^DM/DM^ mice than in tumours grown in ECCre−;FAK^DM/DM^ controls, correlating with the reduced blood vessel density observed in the tumours in ECCre+;FAK^DM/DM^ mice (Figure 5C). In contrast to the results from the ECCre+;FAK^KD/KD^ mice, blood vessel leakage of Hoechst dye was no different in ECCre+;FAK^DM/DM^ mice from that in ECCre−;FAK^DM/DM^ controls (Figure 5D), suggesting that the effect of the FAK KD mutation was overcome by the additional FAK Y397E mutation.

**Figure 6:**
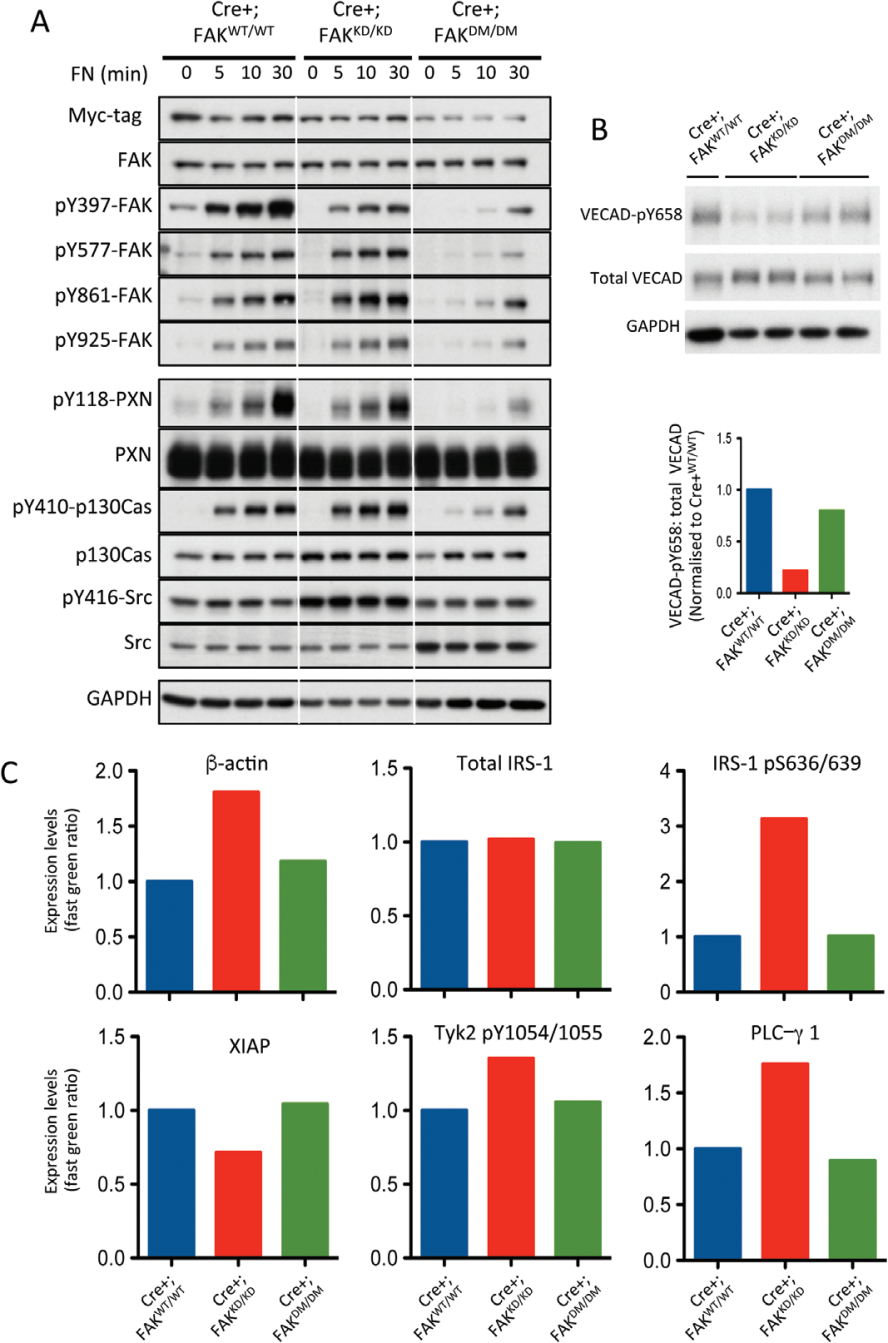
The EC FAK Y397E KD double mutation results in dual FAK and Src kinase inhibition and restores VECAD‐pY658 levels and permeability‐related signalling as compared with Cre+;FAK^KD/KD^ ECs. (A) Cre+;FAK^WT/WT^, Cre+;FAK^KD/KD^ and Cre+;FAK^DM/DM^ ECs were allowed to adhere to fibronectin (FN) for 0, 5, 10 and 30 min, lysed, and analysed by western blotting for levels of myc‐tag, FAK, pY397‐FAK, pY577‐FAK, pY861‐FAK, pY925‐FAK, pY118‐paxillin, paxillin, pY410‐p130Cas, p130Cas, pY416‐Src, and Src. Glyceraldehyde‐3‐phosphate dehydrogenase was used as a loading control. Blots are representative of three experimental repeats. (B) Western blot analysis of Cre+;FAK^WT/WT^, Cre+;FAK^KD/KD^ and Cre+;FAK^DM/DM^ EC lysates for VECAD‐pY658, total VECAD, and GAPDH as a loading control. The bar chart represents mean densitometric readings for duplicate samples. (C) Cre+;FAK^WT/WT^, Cre+;FAK^KD/KD^ and Cre+;FAK^DM/DM^ EC lysates were subjected to RPPA analysis, and expression levels of β‐actin, total IRS‐1, IRS‐1‐pS636/639, XIAP, Tyk2‐pY1054/1055 and PLCγ1 are shown. Bars represent mean values from two experimental repeats.

To begin to explain the changes in tumour growth and tumour blood vessels seen in ECCre+;FAK^KD/KD^ and ECCre+;FAK^DM/DM^ mice, we analysed ECs from these mice. Cre+;FAK^WT/WT^, Cre+;FAK^KD/KD^ and Cre+;FAK^DM/DM^ ECs were trypsinized and allowed to attach to fibronectin for 5, 10 or 30 min, lysed, and subjected to Western blotting. As expected, attachment to fibronectin of Cre+;FAK^WT/WT^ ECs induced phosphorylation of FAK on Y397, Y577, Y861, and Y925, and subsequent phosphorylation of the FAK/Src substrates paxillin and p130Cas. Surprisingly, we detected no fibronectin adhesion‐dependent increase in phosphorylation of Src in this context. However, increased phosphorylation levels of direct Src substrates, such as FAK‐Y577, FAK‐Y861, and FAK‐Y925, as well as p130Cas, clearly demonstrated that its kinase activity was stimulated. Cre+;FAK^KD/KD^ ECs showed reduced phosphorylation of the FAK kinase target FAK‐Y397 as expected, whereas sites of Src‐dependent phosphorylation were unaffected, including FAK‐Y577, FAK‐Y861, FAK‐Y925, and p130Cas‐Y410. Introduction of the Y397E mutation into the DM FAK resulted in a super‐inhibited form, and all the substrates tested showed greatly reduced phosphorylation, indicating that both FAK and Src activities were absent in these cells (Figure 6A). Phosphorylation of targets further down the signalling pathways, such as Akt and JNK1/2, was largely unaffected by either FAK mutant (supplementary material, Figure S2). Together, these data show a rather global effect on signalling, which does not explain why ECCre+;FAK^DM/DM^ mice showed restored vascular leakage as compared with ECCre+;FAK^KD/KD^ mice.

Probably the most studied regulator of vascular permeability is VECAD. Previous work has shown that KD FAK inhibits VECAD Y658 phosphorylation *in vivo* and that this is correlated with reduced vascular leakage 7. Western blot analysis of Cre+;FAK^KD/KD^ ECs revealed reduced VECAD‐pY658 levels as compared with Cre+;FAK^WT/WT^ and Cre+;FAK^DM/DM^ ECs, suggesting that the VECAD‐pY658 regulation correlates with the reduced leakage in ECCre+;FAK^KD/KD^ mice and rescued the leakage phenotype in ECCre+;FAK^DM/DM^ mice (Figure 6B). To examine whether the signalling players involved in permeability also correlated with the reduced leakage in ECCre+;FAK^KD/KD^ mice, we took a non‐candidate approach in which Cre+;FAK^WT/WT^, Cre+;FAK^KD/KD^ and Cre+;FAK^DM/DM^ ECs were subjected to reverse‐phase protein array (RPPA) analysis for 135 different analytes 19. The expression or activity of several molecules was altered in Cre+;FAK^KD/KD^ ECs but not in Cre+;FAK^WT/WT^ or Cre+;FAK^DM/DM^ ECs. These included β‐actin, phospho‐S636/639‐IRS‐1 (but not total IRS‐1 levels), XIAP, Tyk2 pY1054/1055, and PLC‐γ1, all of which are known to be mediators of Src signalling and regulators of permeability 20, 21, 22, 23, 24, 25, 26, 27 (Figure 6C). No changes in phosphorylation of VEGF receptor 2, another regulator of vascular permeability, were observed between any of the genotypes (supplementary material, Figure S3), suggesting that this receptor was not involved in the changes in vascular permeability observed in the Cre+;FAK^KD/KD^ or Cre+;FAK^DM/DM^ mice. Together, these data suggest that the molecular pathways that regulate permeability are altered in Cre+;FAK^KD/KD^ ECs and restored in Cre+;FAK^DM/DM^ ECs.

## Discussion

We showed previously that endothelial FAK is involved in tumour growth and angiogenesis 2. However, genetic ablation of FAK did not allow us to determine whether this was due to FAK kinase or scaffolding activities. Our present data indicate that loss of FAK kinase activity in ECs leads to a similar phenotype as the total loss of FAK in ECs, regarding tumour formation, tumour angiogenesis, and tumour hypoxia 2. This reveals the predominant role of FAK intrinsic kinase activity over its scaffolding functions in tumour growth. Interestingly, the Y397E substitution, designed as a phosphomimetic, was not able to rescue the tumour phenotype observed. However, the structures of phosphotyrosine and glutamate are very different, and it has been proposed before that there is no suitable phosphotyrosine phosphomimetic mutation 28. The presence of glutamate at position Y397 of FAK may therefore not allow the binding and activation of Src and other FAK binding partners, which may explain why this mutant was unable to correct the KD phenotype. To explore this further, we investigated the phosphorylation of FAK and Src‐dependent substrates in response to fibronectin attachment, a well‐known FAK/Src stimulus 29. However, we observed an increase in Src phosphorylation in our immortalized FAK KD ECs as compared with FAK WT cells. This has previously been observed in immortalized MEF KD cells but not in primary cells 12. We also observed high basal levels of phospho‐Src in WT cells, also possibly due to the immortalization, making phospho‐Src an unreliable target to follow. We therefore explored the phosphorylation status of FAK‐Y397, which depends on FAK kinase activity, and known Src targets 30, 31, 32, 33, 34. Consistent with previous results, we observed no inhibition of Src activity in the FAK KD ECs 6 and only a reduction in pY397‐FAK levels, confirming previous results showing that Src is able to phosphorylate this site independently of FAK kinase activity 35. In the FAK DM ECs however, we observed a profound reduction in phosphorylation of all targets. *In vitro,* the DM ECs behaved very similarly to ECs expressing a mutated form of FAK in which exon 15, which codes for 19 amino acids, including Y397, was deleted (FAKΔ15) 36. Whereas the *trans*‐autophosphorylation activity and therefore the kinase activity of FAKΔ15 were unaffected, phosphorylation of FAK‐Y576/77 and FAK‐Y925, as well as p130Cas and paxillin, in response to fibronectin plating was absent in this mutant, similarly to what we observed with the FAK DM ECs. This strongly supports the hypothesis that, in this DM, the glutamate substitution actually profoundly disrupted Src activation. This is in agreement with previous studies showing the importance of Y397 as a binding site for Src, rather than in FAK kinase activation, and the need for both kinases to act as a complex for efficient phosphorylation of substrates 32.

Interestingly, there was a reduction in vascular leakage in the ECCre+;FAK^KD/KD^ mouse tumour vessels that was, however, corrected in the vessels of the ECCre+;FAK^DM/DM^ mice. Both FAK and Src kinases activities have been previously shown to regulate VEGF‐induced permeability *in vivo*, with a reduction in vascular permeability in response to genetic deletion of FAK or Src, or in response to pharmacological inhibitors of these targets 6, 37. Surprisingly, in our study, inhibition of both kinases together reversed this decreased permeability. This suggests that, whereas inhibition of either kinase alone is sufficient to modulate EC barrier strengthening, Src and FAK probably act as a complex in this process, and either need each other's kinase activity or, as has been suggested in previous reports, regulate each other's intracellular localization and thereby downstream signalling 7, 32, 38. Additionally, we show that VECAD phosphorylation at Y658 is downregulated in Cre+;FAK^KD/KD^ ECs and rescued in Cre+;FAK^DM/DM^ ECs. This correlates with reduced leakage in the Cre+;FAK^KD/KD^ mice, and is accompanied by alterations in the levels of β‐actin, IRS‐1‐pS636/639, XIAP, Tyk2 pY1054/1055 and PLCγ1 in Cre+;FAK^KD/KD^ ECs, but not in Cre+;FAK^DM/DM^ ECs, as compared with Cre+;FAK^WT/WT^ ECs. Indeed, these molecules are known to play a role in Src signalling and vessel permeability 20, 21, 22, 23, 24, 25, 26, 27, and the changes observed correlate with reduced leakage in ECCre+;FAK^KD/KD^ mice that is rescued in ECCre+;FAK^DM/DM^ mice. Together, these results suggest that FAK kinase activity and Src work in concert to control VECAD activity and upstream molecular pathways.

Our data regarding tumour formation differ from the data in a 2014 study 7, in which it was shown that FAK KD expression in the endothelium leads to reduced metastasis without affecting primary tumour growth. One should, however, keep in mind the differences between the mouse models used in these studies, as others have used a system in which FAK mutants are expressed in a hemizygous state, whereas, in our current study, FAK mutants are expressed by both alleles. The role of FAK as a dose‐dependent regulator of angiogenesis has previously been stressed. FAK heterozygous mice do not phenocopy WT FAK mice, and, in fact, show enhanced tumour angiogenesis and tumour growth 9. Thus, as the mice in the above study 7 are hemizygous for WT or KD FAK (and not homozygous, as ours are), this may explain the difference in the results.

We have used EC‐specific FAK knockin mutants homozygously expressed to help determine the roles of the kinase domain and Y397 residue *in vivo* in tumour growth and angiogenesis. Overall, our data show that inhibiting FAK kinase activity in ECs is sufficient to reduce tumour growth and angiogenesis, and strongly support the use of FAK inhibitors in the clinic 39. The reduction in vascular leakage observed in ECCre+;FAK^KD/KD^ mice suggests that these inhibitors might have additional positive effects *in vivo*, as they would be expected to reduce vascular permeability, and therefore improve normalization and drug delivery, and also potentially reduce metastasis 7, 40. Outside the context of oncology, it would be interesting to test the effects of FAK kinase inhibitors in ischaemic tissues, where VEGF‐induced permeability has deleterious consequences, exacerbating tissue injury and infarct size 40. The use of other knockin mutants should help to further delineate FAK signalling in physiological and pathological processes *in vivo*.

## Author contributions statement

The authors contributed in the following way: AA: helped to develop the mice, designed, executed and led the experiments, and helped to develop cell lines; DL: assisted with *in vivo* experiments, helped to develop cell lines, carried out the signalling Western blot analysis, and performed the blood vessel density counts; NB: helped to develop and characterize the cell lines, assisted with *in vivo* experiments, and performed the leakage analysis; TL: assisted with *in vivo* experiments, and analysed the pericyte coverage and hypoxia; IF: helped to develop and characterize the cell lines; MD, GD'A: assisted with confocal image acquisition and analysed the perfusion; IF, GD'A: managed the breeding colonies; SB, BT: provided the concept and characterization of the mutant mice; BS: provided the Y397E construct and performed the RPPA analysis; KH‐D: supervised the study; AA, DL, KH‐D: interpreted the data and wrote the paper together.

**SUPPLEMENTARY MATERIAL ONLINE**

**Supplementary materials and methods**

**Supplementary figure legends**

**Figure S1.** Immunofluorescence analysis demonstrates myc‐tag and FAK co‐localisation in cultured Cre+;FAK^WT/WT^, Cre+;FAK^KD/KD^ and Cre+;FAK^DM/DM^ ECs

**Figure S2.** No apparent changes in Akt, JNK1/2 or ERK1/2 levels or phosphorylation in Cre+;FAK^WT/WT^, Cre+;FAK^KD/KD^ and Cre+;FAK^DM/DM^ ECs

**Figure S3.** No apparent changes in VEGF‐receptor 2 in Cre+;FAK^WT/WT^, Cre+;FAK^KD/KD^ and Cre+;FAK^DM/DM^ ECs

## Supporting information


**Supplementary materials and methods**

**Supplementary figure legends**

**Figure S1. Immunofluorescence analysis demonstrates myc‐tag and FAK co‐localisation in cultured Cre+;FAK^WT/WT^, Cre+;FAK^KD/KD^ and Cre+;FAK^DM/DM^ ECs.** Cre+;FAK^WT/WT^, Cre+;FAK^KD/KD^ and Cre+;FAK^DM/DM^ ECs were double‐immunostained for myc‐tag and FAK. Merged images demonstrate that the mutant myc‐tagged FAK co‐localises with FAK in these knock‐in cells. DAPI, blue nuclear marker in merge only. Scale bar: 10 µm.

**Figure S2. No apparent changes in Akt, JNK1/2 or ERK1/2 levels or phosphorylation in Cre+;FAK^WT/WT^, Cre+;FAK^KD/KD^ and Cre+;FAK^DM/DM^ ECs.** Cre+;FAK^WT/WT^, Cre+;FAK^KD/KD^ and Cre+;FAK^DM/DM^ ECs were allowed to adhere to fibronectin (FN) for 0, 5, 10 and 30 mins, lysed and analysed by western blotting for levels of pS473‐Akt, Akt, and p‐JNK1/2, JNK1/2. No apparent differences between genotypes were observed. n = 3 experimental repeats.

**Figure S3. No apparent changes in VEGF‐receptor 2 in Cre+;FAK^WT/WT^, Cre+;FAK^KD/KD^ and Cre+;FAK^DM/DM^ ECs.** Cre+;FAK^WT/WT^, Cre+;FAK^KD/KD^ and Cre+;FAK^DM/DM^ ECs were analysed by reverse phase protein array for levels of VEGFR2‐pY951, pY1059 and pY1175. No differences between genotypes were observed. Bars represent mean values from two experimental repeats.
