# Supplementary material for: Focal Adhesion Kinase (FAK) tyrosine 397E mutation restores the vascular leakage defect in endothelium‐specific FAK‐kinase dead mice
Source: J Pathol. 2017 Jun 1;242(3):358–70. doi: 10.1002/path.4911 (PMC5518444; doi:10.1002/path.4911)
Supplement: Supplementary file 1 — Supplementary materials and methods [file PATH-242-358-s001.docx]

**Supplementary materials and methods**

Reference numbers refer to the main text list

**Mice**

The work described was approved by the QMUL Ethics Committee

PDGFB is predominantly expressed in endothelial cells. The efficiency and endothelial cell-specificity of the Cre-recombinase regulated under this promoter has previously been demonstrated [41-43]**.**

In order to study the effect of inducible endothelial cell-specific FAK mutations *in vivo* we utilized a knockout/knockin system whereby endogenous mouse FAK was deleted and myc-tagged chicken FAK (wild type or mutant) expressed under the control of the Rosa26 (R26) promoter. FAK sequences inserted at the R26 locus were preceded by a loxP-STOP-loxP cassette to allow tamoxifen-induced FAK-knockin expression [10]. Thus we developed Pdgfb-iCre^ert^;FAK^fl/fl^;R26FAK^KD/KD^ mice (FAK-kinase dead (KD), where the lysine at residue 454 of the ATP binding site has been mutated to an arginine) and Pdgbf-iCre^ert^;FAK^fl/fl^;R26FAK^DM/DM^ mice (double mutant (DM): KD mutation with a Y397E mutation, where the tyrosine 397 has been mutated to a glutamate). All mice were homozygous for both FAK^fl/fl^ and R26FAK-knockin genes. Pdgfb-iCre^ert^;FAK^fl/fl^;R26FAK^WT/WT^ mice expressed wild type FAK and were used to validate the system. Pdgfb-iCre^ert^-negative littermates acted as controls. Tamoxifen treatment induced endothelial cell-specific endogenous mouse FAK deletion and knockin-FAK expression only in those mice expressing Pdgfb-iCre^ert^.

**PCR genotyping**

Genotyping for Pdgfb-iCre^ert^; FAKfloxed allele and Rosa26 targeting was performed in ear snipped digested DNA. For primer sequences see Tavora *et al*. [10].

**Reverse Transcription – quantitative PCR (RT-qPCR)**

RNA was extracted from ECs and mouse hearts. For cells, an RNA extraction kit (Qiagen) was used. Mouse heart RNA was extracted using Trizol reagent (Life Technologies). A high capacity cDNA reverse transcription kit (Applied Biosystems) and custom made FAK mouse and chicken specific primers and probes for real time PCR Taqman reactions were obtained from Applied Biosystems. The primers and probes sequences are described in Tavora *et al*. [10].

**RIPA lysates from hearts**

Hearts from mice used in the tumour growth experiments were frozen on dry ice then crushed in liquid nitrogen using a pestle and mortar. Protein was extracted in 1ml RIPA buffer per heart. Lysates were syringed and were then centrifuged at 10,000g for 10 min. The clear supernatants were transferred into a clean tube and stored until analysis.

**Western blotting**

Aliquots (11μg) of total protein lysates were separated by reducing SDS-PAGE and transferred onto nitrocellulose membranes. The membranes were blocked for 30 min at RT in 5% milk in Tris buffered saline + 0.1% Tween-20 (TBST) then incubated overnight at 4 °C in 4% BSA in TBST with the following antibodies: myc-tag clone 9E10 (Abcam), pY397-FAK (#3283), pY925-FAK (#3285), pY410-p130Cas (#4011), pY416-Src (#2101) from Cell Signaling Technology, pY577-FAK (#44614G), pY118-Paxillin (#44722G) from Invitrogen, pY861-FAK (#PS1008), pY658-VECAD [44], GAPDH (#MAB374) from Millipore, and with HRP-conjugated sheep anti-mouse or goat anti-rabbit (Jackson Immunoresearch). The membranes were stripped and reprobed for total protein levels: FAK (#610088), Paxillin (#610052), VE-Cadherin (#550548) from BD Transduction Laboratories, p130Cas (#05-469, Millipore), Src (#2110) from Cell Signaling Technology.

**Reverse phase protein array (RPPA) Nitrocellulose slides**

Cell lysates were prepared using ice cold lysis buffer (1% Triton X-100, 50 mM HEPES [pH 7.4], 150 mM NaCl, 1.5 mM MgCl_2_, 1 mM EGTA, 100 mM NaF, 10 mM NaPPi, 10% glycerol, supplemented with Complete Protease Inhibitor tablets (Boehringer/Roche, Mannheim, Germany) and 1 mM Na_3_ VO_4_).

After normalisation of protein concentrations, triplicate spots of each lysate were deposited onto 16-pad Avid Nitrocellulose slides (Grace Bio) under conditions of constant 70% humidity using an Aushon 2470 Array platform (Aushon BioSystems). After printing and washing the arrays were blocked by incubation in Superblock (Thermo Scientific #37535) for 10 min. The protein array chips were subsequently incubated for 1 h with primary antibody followed by repeat blocking with Superblock and a 30 min incubation with anti-rabbit Dylight-800 conjugated secondary antibody (Cell Signaling Technology # 5151).

Following secondary antibody incubation and subsequent wash steps the immune-stained arrays were imaged using an Innopsys 710IR scanner (Innopsys France). Microarray images were obtained at the highest gain without saturation of fluorescent signal detection. Image analysis was performed using Mapix software (Innopsys France) to calculate the relative fluorescence intensity (RFI) value for each sample. An estimate of total protein printed per feature on the array was determined by staining an array slide with fast green protein stain. Readout values for all antibodies tested are expressed as a ratio of total protein loaded and are presented as the mean of technical replicates.
