# Supplementary material for: Focal Adhesion Kinase (FAK) tyrosine 397E mutation restores the vascular leakage defect in endothelium‐specific FAK‐kinase dead mice
Source: J Pathol. 2017 Jun 1;242(3):358–70. doi: 10.1002/path.4911 (PMC5518444; doi:10.1002/path.4911)
Supplement: Supplementary file 2 — Supplementary figure legends [file PATH-242-358-s002.doc]

**Supplementary figure legends**

**Figure S1. Immunofluorescence analysis demonstrates myc-tag and FAK co-localisation in cultured Cre+;FAK^WT/WT^, Cre+;FAK^KD/KD^ and Cre+;FAK^DM/DM^ ECs.** Cre+;FAK^WT/WT^, Cre+;FAK^KD/KD^ and Cre+;FAK^DM/DM^ ECs were double-immunostained for myc-tag and FAK. Merged images demonstrate that the mutant myc-tagged FAK co-localises with FAK in these knock-in cells. DAPI, blue nuclear marker in merge only. Scale bar: 10 μm.

**Figure S2. No apparent changes in Akt, JNK1/2 or ERK1/2 levels or phosphorylation in Cre+;FAK^WT/WT^, Cre+;FAK^KD/KD^ and Cre+;FAK^DM/DM^ ECs.** Cre+;FAK^WT/WT^, Cre+;FAK^KD/KD^ and Cre+;FAK^DM/DM^ ECs were allowed to adhere to fibronectin (FN) for 0, 5, 10 and 30 mins, lysed and analysed by western blotting for levels of pS473-Akt, Akt, and p-JNK1/2, JNK1/2. No apparent differences between genotypes were observed. n=3 experimental repeats.

**Figure S3.** **No apparent changes in VEGF-receptor 2 in Cre+;FAK^WT/WT^, Cre+;FAK^KD/KD^ and Cre+;FAK^DM/DM^ ECs.** Cre+;FAK^WT/WT^, Cre+;FAK^KD/KD^ and Cre+;FAK^DM/DM^ ECs were analysed by reverse phase protein array for levels of VEGFR2-pY951, pY1059 and pY1175. No differences between genotypes were observed. Bars represent mean values from two experimental repeats.
