# Supplementary figures and images for: Focal Adhesion Kinase (FAK) tyrosine 397E mutation restores the vascular leakage defect in endothelium‐specific FAK‐kinase dead mice
Source: J Pathol. 2017 Jun 1;242(3):358–70. doi: 10.1002/path.4911 (PMC5518444; doi:10.1002/path.4911)

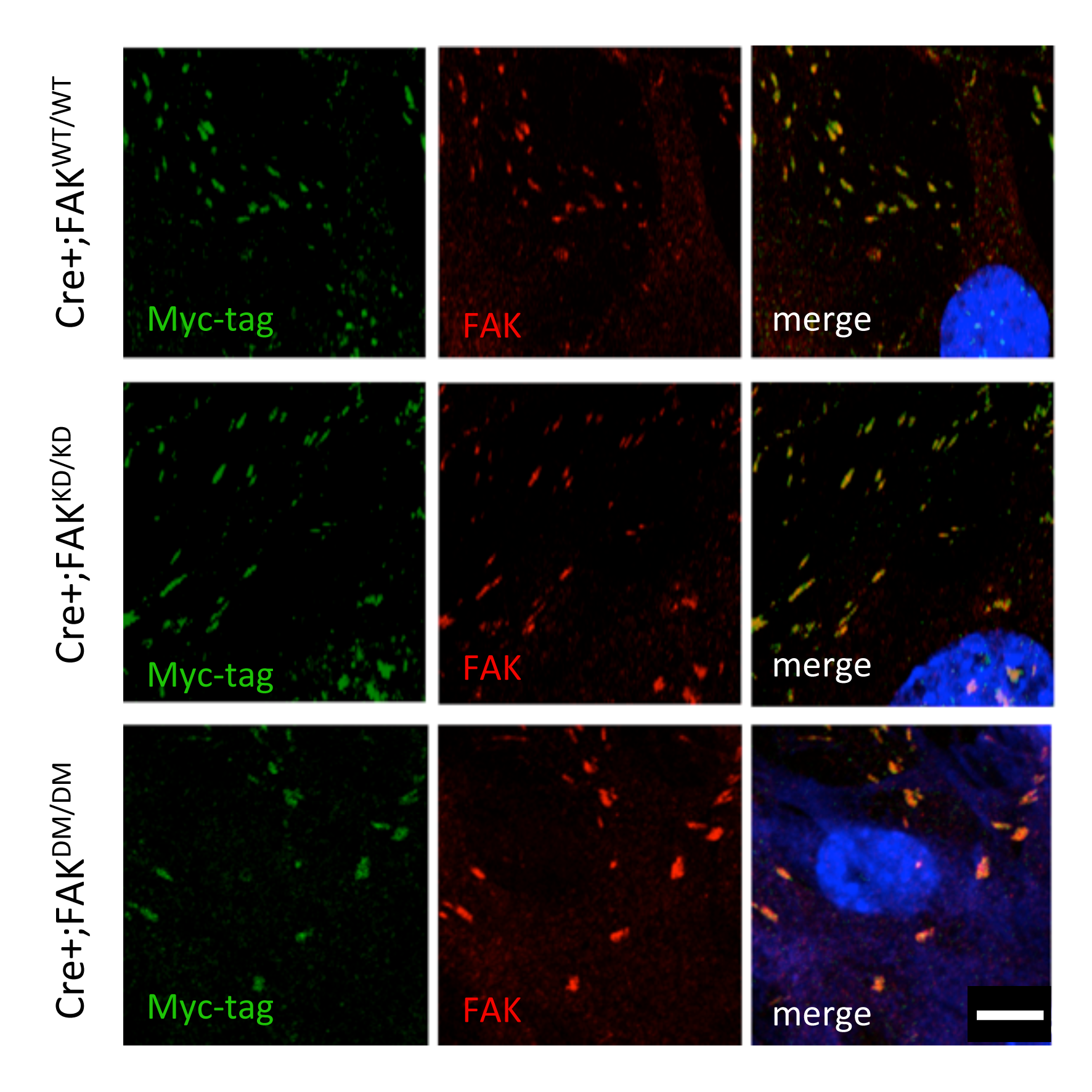

Supplement: Supplementary file 3 — Figure S1. Immunofluorescence analysis demonstrates myc‐tag and FAK co‐localisation in cultured Cre+;FAKWT/WT, Cre+;FAKKD/KD and Cre+;FAKDM/DM ECs. Cre+;FAKWT/WT, Cre+;FAKKD/KD and Cre+;FAKDM/DM ECs were double‐immunostained for myc‐tag and FAK. Merged images demonstrate that the mutant myc‐tagged FAK co‐localises with FAK in these knock‐in cells. DAPI, blue nuclear marker in merge only. Scale bar: 10 µm. [file PATH-242-358-s003.tif]

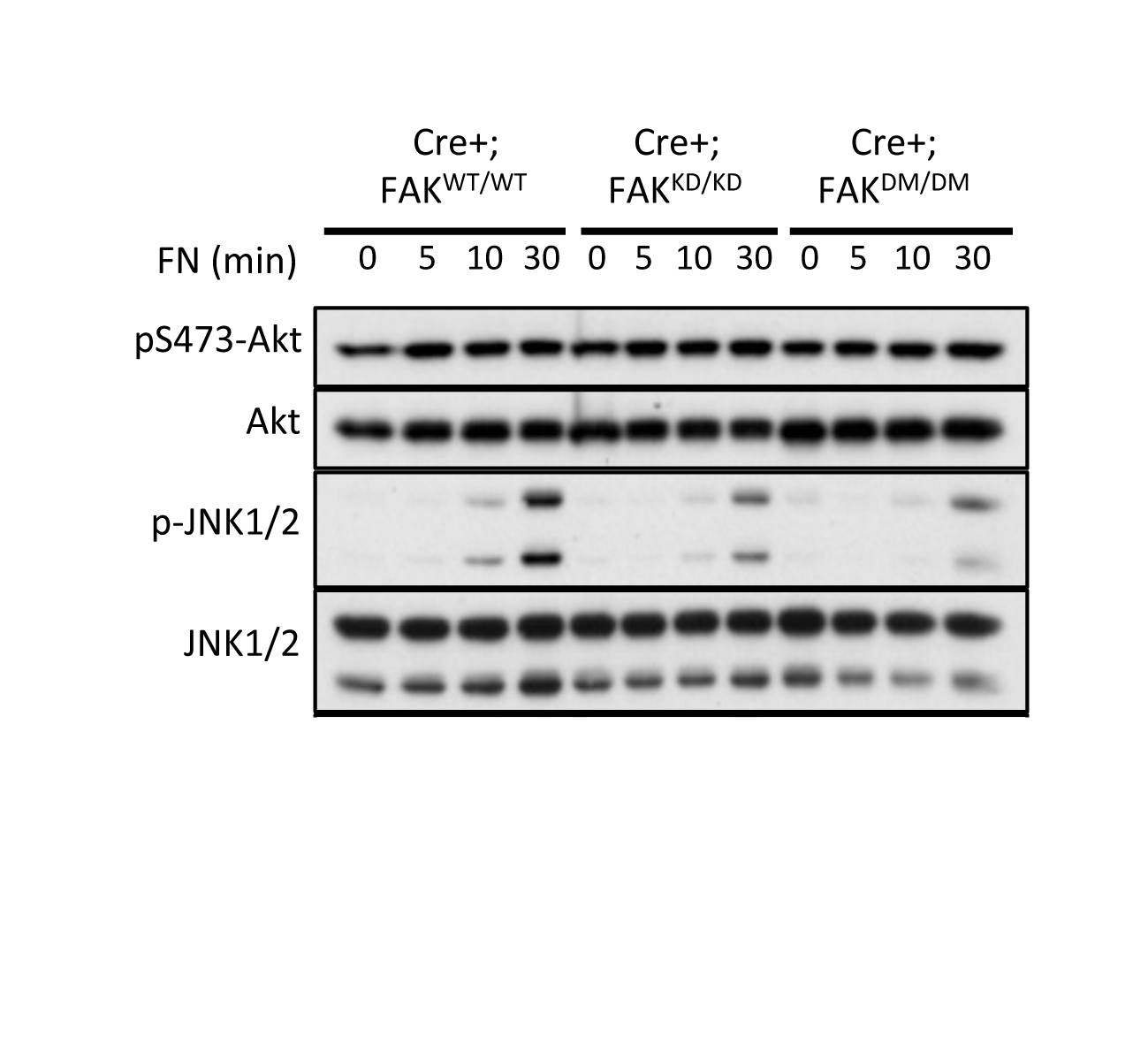

Supplement: Supplementary file 4 — Figure S2. No apparent changes in Akt, JNK1/2 or ERK1/2 levels or phosphorylation in Cre+;FAKWT/WT, Cre+;FAKKD/KD and Cre+;FAKDM/DM ECs. Cre+;FAKWT/WT, Cre+;FAKKD/KD and Cre+;FAKDM/DM ECs were allowed to adhere to fibronectin (FN) for 0, 5, 10 and 30 mins, lysed and analysed by western blotting for levels of pS473‐Akt, Akt, and p‐JNK1/2, JNK1/2. No apparent differences between genotypes were observed. n = 3 experimental repeats. [file PATH-242-358-s004.tif]

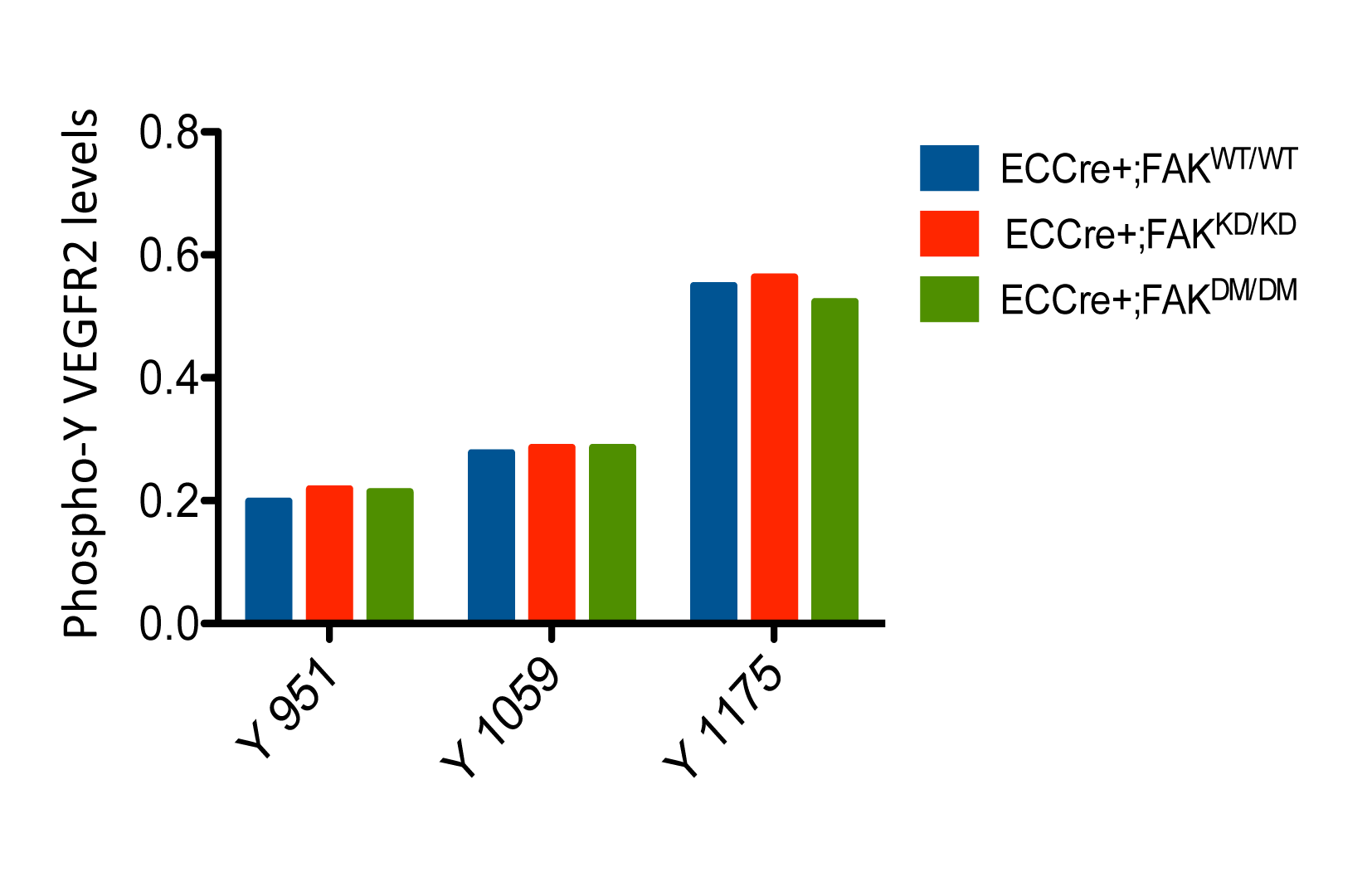

Supplement: Supplementary file 5 — Figure S3. No apparent changes in VEGF‐receptor 2 in Cre+;FAKWT/WT, Cre+;FAKKD/KD and Cre+;FAKDM/DM ECs. Cre+;FAKWT/WT, Cre+;FAKKD/KD and Cre+;FAKDM/DM ECs were analysed by reverse phase protein array for levels of VEGFR2‐pY951, pY1059 and pY1175. No differences between genotypes were observed. Bars represent mean values from two experimental repeats. [file PATH-242-358-s005.tif]
